# Temporal and Spatiotemporal Arboviruses Forecasting by Machine Learning: A Systematic Review

**DOI:** 10.3389/fpubh.2022.900077

**Published:** 2022-06-03

**Authors:** Clarisse Lins de Lima, Ana Clara Gomes da Silva, Giselle Machado Magalhães Moreno, Cecilia Cordeiro da Silva, Anwar Musah, Aisha Aldosery, Livia Dutra, Tercio Ambrizzi, Iuri V. G. Borges, Merve Tunali, Selma Basibuyuk, Orhan Yenigün, Tiago Lima Massoni, Ella Browning, Kate Jones, Luiza Campos, Patty Kostkova, Abel Guilhermino da Silva Filho, Wellington Pinheiro dos Santos

**Affiliations:** ^1^Nucleus for Computer Engineering, Polytechnique School of the University of Pernambuco, Poli-UPE, Recife, Brazil; ^2^Department of Atmospheric Sciences, IAG-USP, University of São Paulo, São Paulo, Brazil; ^3^Center for Informatics, Federal University of Pernambuco, CIn-UFPE, Recife, Brazil; ^4^Centre for Digital Public Health and Emergencies, Institute for Risk and Disaster Reduction, University College London, London, United Kingdom; ^5^Boǧaziçi University, Institute of Environmental Sciences, Istanbul, Turkey; ^6^Department of Systems and Computing, Federal University of Campina Grande, Campina Grande, Brazil; ^7^Centre for Biodiversity and Environment Research, Department of Genetics, Evolution and Environment, University College London, London, United Kingdom; ^8^Department of Civil Environmental and Geomatic Engineering, University College London, London, United Kingdom; ^9^Department of Biomedical Engineering, Federal University of Pernambuco, DEBM-UFPE, Recife, Brazil

**Keywords:** digital epidemiology, computational intelligence, arboviruses forecast, machine learning, systematic review, dengue, chikungunya, Zika virus

## Abstract

Arboviruses are a group of diseases that are transmitted by an arthropod vector. Since they are part of the Neglected Tropical Diseases that pose several public health challenges for countries around the world. The arboviruses' dynamics are governed by a combination of climatic, environmental, and human mobility factors. Arboviruses prediction models can be a support tool for decision-making by public health agents. In this study, we propose a systematic literature review to identify arboviruses prediction models, as well as models for their transmitter vector dynamics. To carry out this review, we searched reputable scientific bases such as IEE Xplore, PubMed, Science Direct, Springer Link, and Scopus. We search for studies published between the years 2015 and 2020, using a search string. A total of 429 articles were returned, however, after filtering by exclusion and inclusion criteria, 139 were included. Through this systematic review, it was possible to identify the challenges present in the construction of arboviruses prediction models, as well as the existing gap in the construction of spatiotemporal models.

## 1. Introduction

Vector-borne diseases present a major public health challenge for many countries around the world (1–3). Arboviral diseases are diseases caused by arthropod-borne viruses which are viruses that need a vertebrate host and a hematophagus arthropod (the transmitting vector) in order to maintain themselves in nature (4–6). Arboviruses transmitted by *Aedes aegypti*, e.g., manage to maintain themselves in nature through a human-mosquito cycle. In other words, for the transmission of one of these diseases, it is only necessary for the hematophagous arthropod to inject its infectious saliva into the blood of a non-viremic individual at the time of the bite. However, non-vertical transmission is also possible, such as during sexual intercourse, from mother to child during pregnancy or childbirth, in addition to transmission of blood, bone marrow, and organ transplantation (6).

Since arboviruses are part of the Neglected Tropical Diseases (NTDs) group, they impact directly and indirectly the countries wherein they are endemic (7). The direct impact is related to the number of people infected and the number of deaths caused by arboviruses. On the other hand, the indirect impact is more associated with socioeconomic impacts (7). Dengue, Zika, and chikungunya fever, transmitted by Aedes mosquitoes, are examples of diseases that belong to the group of NTDs. According to the World Health Organization, dengue fever is present in more than 100 countries around the world. Furthermore, in the last decade, there has been an increase of around 300% in the number of cases of the disease (2). Chikungunya, in turn, has been identified in more than 60 countries since 2004, when it first spread to countries in Europe and the Americas (8), whereas the Zika virus is currently present in a total of 86 territories around the world (9). Thus, the arboviral diseases rapid global spread amplified the challenges faced by the scientific and governmental communities (10).

The arboviruses dynamics are associated with several heterogeneous factors that involve demographic, climatic, and environmental aspects of a region. Demographic changes arising from intense migratory flows from rural to urban areas have led cities to grow inordinately. The swelling of urban populations along with urban population mobility associated with other factors, such as poor sanitation, also plays an important role in transmission vector proliferation. In addition, the lack of water distribution, as well as the difficult access to health systems, also bring barriers to controlling the vector (3, 11, 12). Another aspect associated with arbovirus dynamics is the local climatic and environmental conditions. Luminosity, rainfall, relative humidity, and temperature, act directly on the mosquitoes' development and interfere with the eggs' hatch, as well as their lifetime and dispersion (3, 11, 13).

With climate change and the increase in the number and frequency of international flights, two new arboviruses transmitted by the *A. aegypti* mosquito have emerged in Brazil: Chikungunya and the Zika virus. Raising, in this way, new challenges regarding the control and monitoring of the vector (14–19).

Hence, considering the impact caused by the vector-borne diseases, several research groups have directed their efforts to understand the dynamics of arboviruses through mathematical and computational models for the creation of prediction models (3, 20, 21). We believe that prediction models can be a good tool for health authorities to implement public policies for rapid monitoring and control of the arboviruses spread. Therefore, this document proposes a systematic review of the literature to identify models for predicting arboviruses cases transmitted by the *A. aegypti*—dengue fever, Zika virus disease, and chikungunya—as well as the mosquito dynamics. In particular, this review seeks to answer the following research questions. In particular, this systematic review seeks to analyze what are the biggest challenges when it comes to implementing arboviruses prediction models. In addition, we sought to identify the main techniques for predicting mosquito cases or foci and which are the main variables that interfere in the dynamics of disease transmission and the dynamics of the transmission vector.

## 2. Method

**Figure 1:**
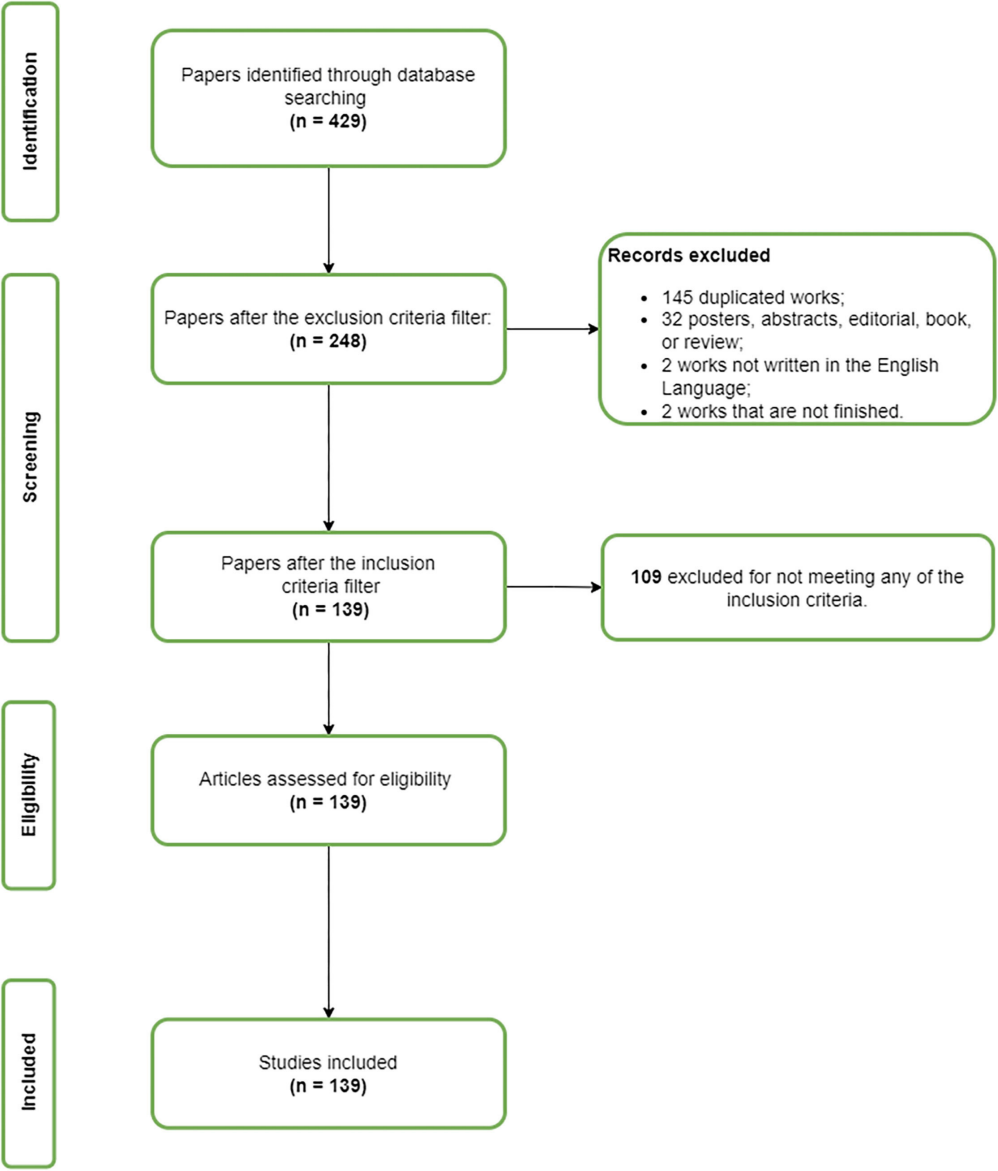
This system consisted of the following steps: (1) First, we performed a search of scientific databases (IEEE Xplore, PubMed, Scopus, Science Direct, and Springer Link). (2) We then filtered the returned articles according to the exclusion criteria. (3) In the next step, we selected the article that remained from the previous stage according to the inclusion criteria. (4) After completing the previous step, we read, evaluated, and summarized the studies included in the review. (5) In the last step of this review, we grouped the studies considering their common characteristics.

The strategy for conducting this systematic review is detailed in Figure 1. First, we performed an automatic search in scientific databases, such as IEE Xplore, PubMed, Science Direct, Springer Link, and Scopus. We searched for articles published between 2015 and 2020 wherein the metadata, titled or abstract contained the terms defined in the following search string: [“Arboviruses” **OR** “arthropod-borne virus” **OR** “dengue” **OR** “chikungunya” **OR** “mosquito-borne disease”] **AND** [“Machine Learning” **OR** “Deep Learning” **OR** “neural network” **OR** “artificial intelligence”] **AND** [“forecast” **OR** “prediction”].

In the following step, we identified the number of articles that were retrieved from each scientific database. We then checked if the articles met the exclusion criteria. In this review, we excluded works that were not in English, works that were not completed, and documents classified as posters, tutorials, editorials, or calls for articles. We also excluded works that did not include arbovirus or breeding site prediction and works that did not include computational techniques.

After filtering according to the exclusion criteria, we briefly read the article's abstract, introduction, and conclusion. This step was essential in order to select the articles according to the inclusion criteria. The works selected in that phase were those which met at least one of the following criteria:

1. Works with computational intelligence methods to predict arboviruses cases.
2. Works with computational intelligence methods to predict mosquito breeding sites.
3. Works with computational intelligence methods to predict the mosquitos' dynamics.
4. Works with statistical learning (Bayesian and other probabilistic methods).
5. Works involving forecasting with differential equations.

The remaining articles after filtering by the inclusion criteria were fully read and evaluated according to the quality criteria described in Table 1. We used a 0-1 scale to assess study quality, where Yes (Y) = 1; Partially (P) = 0.5, and No (N) = 0. Three reviewers performed articles assessment, independently, and the disagreements were resolved by discussion among the reviewers.

**Table 1:** Quality criteria used to evaluate the selected studies.

**ID**	**Quality criteria**	**Answer**
QC1	Are the objectives clearly stated?	Y/P/N
QC2	Are the data sources clearly described?	Y/P/N
QC3	Do the authors present the variables to build their models?	Y/P/N
QC4	Do the author explicitly defined which computational techniques or prediction model they used as well as their architectures and parameters?	Y/P/N
QC5	Do the authors report which metrics they used in order to evaluate their models?	Y/P/N
QC6	Are the conclusions coherent to the study findings and also with the set objectives?	Y/P/N
QC7	Do the authors detail the weakness of their work?	Y/P/N

From the articles selected by the inclusion criteria, we extract the following information: the title of the article, the name of the authors, the institution, the application of the study, the methodology applied to the study, the prediction model, results, the advantages, and the disadvantages of the method.

## 3. Results and Discussion

The search process returned 51 articles from IEEE Xplore, 95 articles from PubMed, 238 from Scopus, 20 from Science Direct, and 25 from Springer Link. It is important to emphasize that, for the Science Direct database, the search string had to be reduced. For this database, the number of Boolean operators in the original string search was not supported. In this case, we used the terms: (“dengue” **OR** “zika” **OR** “chikungunya”) **AND** (“Machine Learning” **OR** “artificial intelligence” **OR** “regression”) **AND** (“forecast” **OR** “prediction”). From the 429 works collected, 181 were excluded in the filtering by the exclusion criteria stage. Among these 181 articles, 145 were duplicated studies, 32 were posters, abstracts, books, proceedings, or systematic literature review. In addition, two of them were excluded because they were not in English, and two articles were unfinished. We then screened the remaining 248 studies by reading the title, abstract, and conclusion. After the inclusion criteria stage, 109 were removed from this study for not meeting any of the inclusion criteria. Hence, 139 articles were included in this systematic review.

In the last step of the systematic review, we grouped the 139 selected articles according to their common characteristics (Table 2). The studies were divided into six groups: Arboviruses (counts) prediction (Group 1), Arboviruses detection (Group 2), Outbreaks and Risk prediction (Group 3), Models of mosquitoes dynamics, breeding sites models (Group 4), Clustering, modeling, and spatiotemporal prediction of arboviruses (Group 5), and Other Approaches (Group 6). In Group 1, we considered only the studies that presented models for counting arboviruses. In Group 2, we only included the studies that involved arboviruses detection. Group 3, in turn, is composed of studies that present models for predicting arbovirus outbreaks, as well as predicting the risk of an outbreak. The studies that presented vector monitoring and prediction models were included in Group 4. Those articles that investigated arboviruses prediction models with a spatiotemporal approach were included in Group 5. Finally, the studies that presented more than one of the approaches mentioned above—or that did not fit into any of the previous groups—were included in Group 6.

**Table 2:** Number of studies per group, considering the following stratification: Group 1: prediction of arboviruses by counting; Group 2: detection of arboviruses; Group 3: prediction of risk and epidemiological outbreaks of arboviruses; Group 4: modeling the dynamics of mosquitoes and breeding sites; Group 5: spatio-temporal modeling; Group 6: other approaches.

**Group**	**Description**	**Number of studies**
Group 1	Arboviruses (count) prediction	80
Group 2	Arboviruses detection	15
Group 3	Arboviruses Outbreaks and Risk prediction	18
Group 4	Models of mosquitoes dynamics, breeding sites models	10
Group 5	Clustering, spatiotemporal modeling	9
Group 6	Other approaches	7

### 3.1. Arboviruses (Counts) Prediction

**Figure 2:**
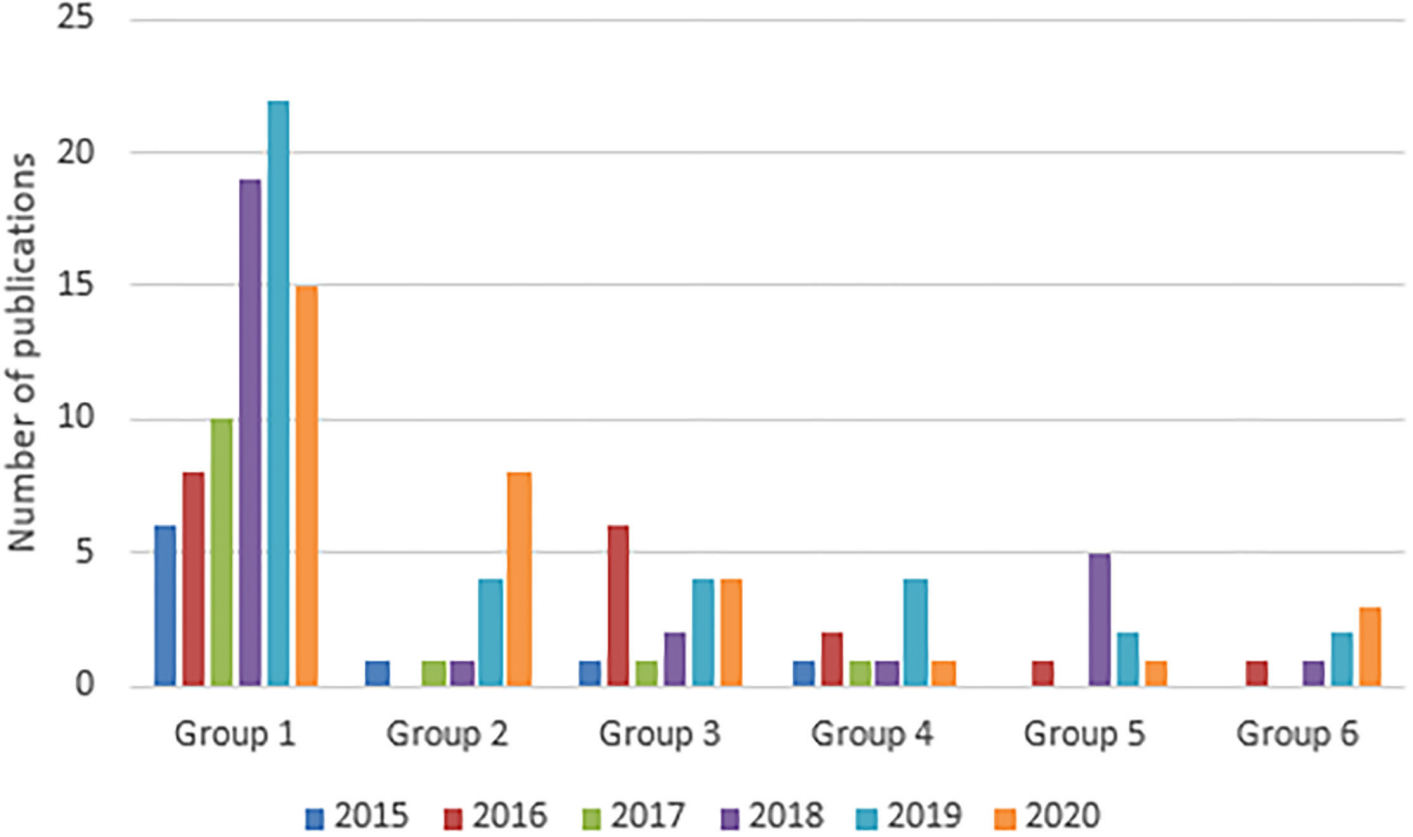
Distribution of the number of articles according to the year of publication for each group.

**Figure 3:**
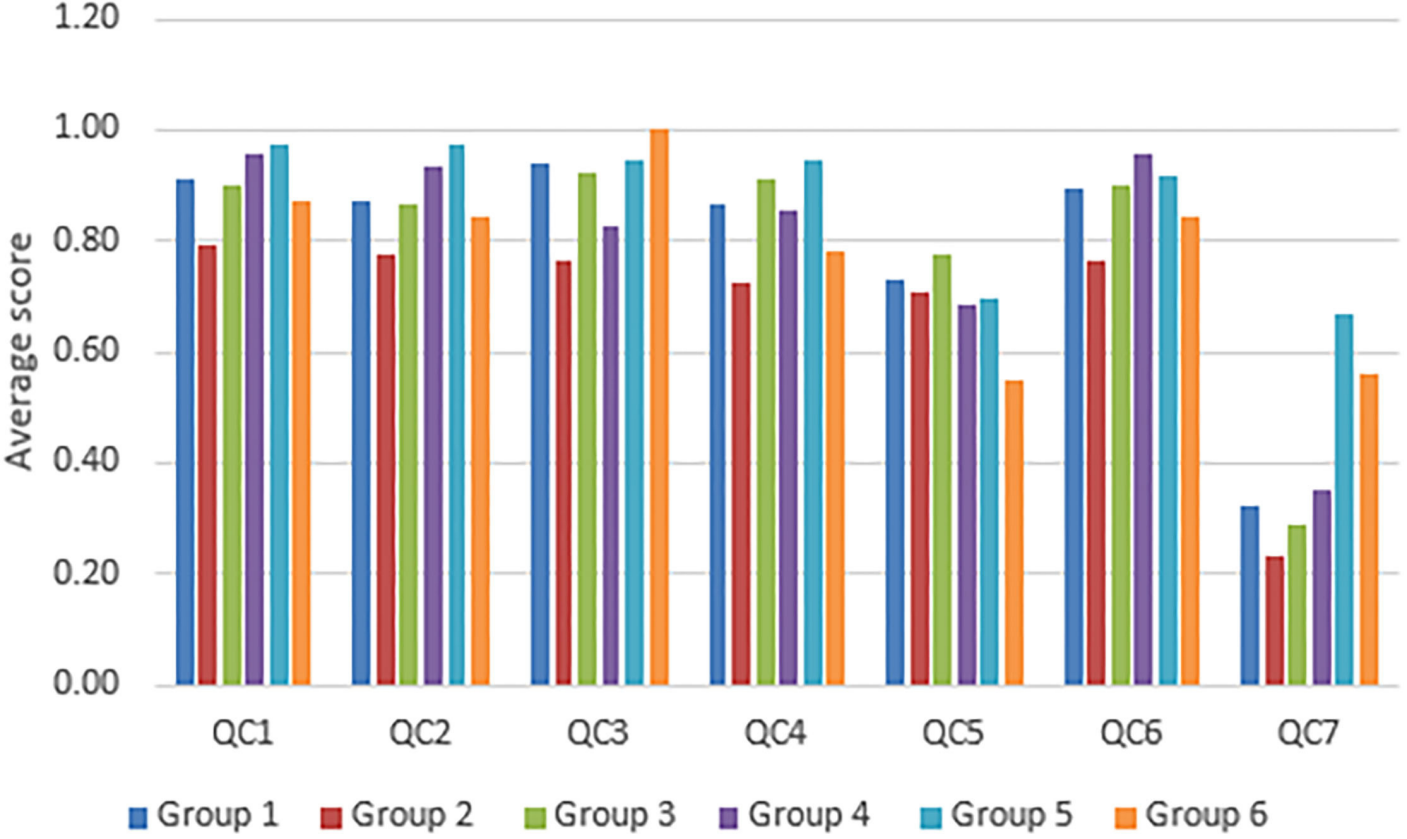
Average score for each quality criteria for the studies from each group.

Among the 139 selected studies, about 80 studies are related to the prediction of the incidence of arboviruses cases (Table 2). Considering the year of publication, we observed that most of the studies in this group were published in 2018 and 2019. The number of articles published in 2018 and 2019 was 19 and 22, respectively, with a drop in the number of publications related to this topic in the year 2020 (Figure 2). Regarding the scores referring to quality criteria, we noticed that most scores were high, with the exception of QC7. For this criterion, the average score achieved by the studies was 0.33 (Figure 3).

The *A. aegypti* is the transmitter vector of three different type arboviral diseases. Taking into account the types of arboviruses transmitted by this mosquito, we found a significant amount of work focused on the construction of dengue fever transmission models. In these studies, the authors, in most cases, do not distinguish the serotype of the disease. In other words, dengue cases are generally considered as: dengue fever, dengue hemorrhagic fever and dengue shock syndrome, including local and imported cases. However, the studies of (22–27) are only focused on prediction models for dengue hemorrhagic fever. Regarding the other two diseases transmitted by the *Ae. aegypti*, we found a small number of articles addressing Zika virus disease and chikungunya's numeric prediction models (28–33).

The returned studies also brought a great variety related to the attributes used to build arboviruses prediction models. It is noted that, in several studies, prediction models are built taking into account only past values of disease cases (17, 25, 29–31, 34–38). However, arboviruses are diseases that need a transmitting vector for the arbovirus cycle in nature to complete. Furthermore, climatic factors directly affect the life cycle of the transmitting mosquito. In this context, several studies have investigated prediction models considering the effect of climatic and environmental variables on arbovirus transmission. Therefore, we observed a wide variety of studies that used at least one of the following variables as model attributes: temperature, rain, and relative humidity. However, some studies included other parameters in their models such as the number of rainy days (39–41), number of stormy days, and wind speed (41).

Furthermore, environmental variables obtained through remote sensing were also explored in a relevant number of studies. The most common were normalized difference vegetation index (NDVI) (42–44), vegetation index (45), enhanced vegetation index (46), smoothed vegetation index, smoothed brightness temperature index, vegetation condition index, vegetation health index (44), land surface temperature (43, 46, 47), Southern Oscillation Index (SOI), and Sea Surface Temperature Anomaly (SSTA) (48). In the studies of (47) and (44), the authors included information on the EL Ninõ phenomenon as well as (47)—that included variables related to the El Niño Southern Oscillation Index—and (44)—that included the Oceanic Niño Index variable in their model.

The research groups also explored attributes other than climate variables, such as epidemiological surveillance variables and sociodemographic variables. Among the epidemiological surveillance variables, the most used were: the number of larva-free, house index (39, 49), weekly breeding percentage (50), container (49), breteau index (49, 51, 52), standard space index, adult mosquito density, *Ae. aegypti* larvae infection, and female mosquito infection rate (52). Mosquito dynamics interfere with arbovirus dynamics. Including this information in predictive models can be an outlet for the search for more robust models that can understand the arboviruses' dynamics in a given region. Sociodemographic aspects also influence the arboviruses dynamics. Considering this fact, Dharmawardana et al. (45) also implemented in their model a mobility model in order to predict the dengue cases's incidence curve. Still considering sociodemographic information, other researchers included in their models' population density (32, 40), poverty percentage (32), population (41, 46, 53), Gini Index—a measure of income inequality—, education coverage (24), and unavailability of the garbage dump. In the model developed by (50), the population size attribute was considered for both the resident population and non-resident foreign population. Models considering sociodemographic factors can help us to understand how population dynamics are related to arboviruses cases. In this way, it can help to guide socio-educational actions and to direct the implementation of basic sanitation and infrastructure policies.

Continuing the analysis regarding the variables included in the prediction models, we observed that data from social media and search volume reported by search engines can be a powerful tool in monitoring arbovirus-borne diseases. In the study of (54), data from Baidu (a popular search tool in China) and social media are used to model the incidence of dengue in Guangzhou, China. Data referring to the number of comments, number of likes, and number of forwarding that are associated with dengue as a primary keyword are captured. In the studies of (30) and (27), the authors use Google Trends data to generate models for predicting Zika and dengue hemorrhagic fever, respectively. Espina and Estuar (36), in turn, use Twitter data to identify infodemiological content to be used in predicting dengue. In a world where information is gaining speed at every moment, the implementation of arbovirus models using social media can be an alternative for monitoring, surveillance, and disease prediction.

Taking into account the datasources used by the authors, we identified that in a vast majority of the returned studies, the data were obtained through government institutions. These institutions were responsible for either epidemiological surveillance or meteorological monitoring of the study area. One of the limitations presented in studies that use government data is the underreporting cases (55). Usually, when the individuals do not have the most severe form of the disease, they do not seek health services. Hence, under these conditions, those individuals are not included in the statistics. Moreover, health data usually have other limitations such as missing values, e.g., (55). However, some works use alternative sources to obtain data. Data can also be obtained through social media and search engines (27, 29, 30, 36, 54) and through data from the WHO (29, 31). On the other hand, we observed that in some studies, the authors do not explicit the origins of the collected data (37, 39, 43, 44, 56–60). The lack of information regarding the datasources can affect the study's reproducibility since the databases' original conditions to generate the models are not clear.

When we evaluated the studies regarding the types of models used in the predictions, we observed that the vast majority of authors investigated moving average models (27), such as the Autoregressive Integrated Moving Average (ARIMA) (17, 23, 29, 35, 41, 43, 46, 56, 61–63), Seasonal Autoregressive Integrated Moving Average (SARIMA) (55, 63–66), Autoregressive Integrated Moving Average with Explanatory Variable (ARIMAX) (67). Several works have also presented a wide variety of models using artificial neural networks, mainly the LSTM (59, 68–70). But models using backpropagation neural networks (BPNN), GANN networks (60), Elman Recurrent Neural Network Levenberg Marquardt Algorithm (ERMN/LMA) (22), and Deep feed-forward neural networks (28) were also investigated. Although neural networks have been extensively explored, in many studies, the authors did not explain the type of network they were investigating (23, 45, 46, 61, 66, 71, 72).

When working with prediction, we also prioritize the computational cost associated with the implemented technique. In this sense, optimization algorithms can help us to reduce the computational cost by reducing model training time. Optimization algorithms do this by looking for attributes that represent the dataset being studied. Therefore, some studies in this group investigated some optimization techniques. In the study of (57), the authors investigated several optimization algorithms associated with the Least Square Support Vector Machine (LSSVM). The investigated algorithms were: Moth Flame Optimization (MFO), Gray Wolf Optimizer (GWO), Firefly Algorithm (FA), and Artificial Bee Colony (ABC) algorithm. Saptarini et al. (22), in turn, used Genetic Algorithm, as well as (68). Finally, we notice that, when it comes to predicting the count or incidence of arboviruses, there are a wide variety of model applications.

In the articles evaluated by this group, we observed that among the main diseases transmitted by *A. aegypti*, the models for predicting dengue cases are the most explored by research groups. As the diseases transmitted by this vector present similar symptoms in their milder forms, in regions where dengue, zika, and chikungunya viruses circulate, the models may present errors related to the low distinction between the diseases. The models that included climatic variables and/or variables of sociodemographic aspects performed better than those that only took into account the historical series of confirmed cases of the disease.

As for the origin of the data sources, we observed that the data obtained by governmental institutions provide greater reliability to the models. However, these models have limitations, mainly in relation to the underreporting of cases. Underreporting can impair the performance of case prediction models. The data that are obtained through the analysis of user behavior in social networks can act as an important tool in the prediction of arboviruses. Since, in some regions, the public system may take a long time to update notifications, alternative data sources can be a good solution in the development of robust models, especially in critical situations such as during arbovirus outbreaks. On the other hand, models generated using data solely from social networks may not be applied in regions where access to the Internet and mobile devices are scarce. That is, in some peripheral regions, the model may not be able to identify disease cases in that region.

Regarding the prediction models selected, we observed that most of the studies that used Artificial Intelligence opted for deep learning models. Despite the promising results that were obtained, the use of deep networks, such as LSTM, is linked to large memory consumption. In other words, it takes a lot of training time and resources to create applications for the real world. Moving average models, in turn, are good tools for capturing trends, periodic changes, and random distortions in historical series. In addition, they are simple and quick to apply.

Thus, the models of the historical series are very relevant and can be a very useful tool in the planning of public policies to combat arboviruses. However, these models are not able to provide information regarding the spatial distribution of diseases. That is, they are not able to point out which areas are being more or less affected by diseases transmitted by *A. aegypti*.

### 3.2. Arboviruses Detection

For this group, 15 studies were selected from the 139 included in this review (Table 2). The publication years for this group varied between 2015 and 2020, wherein the majority of the studies were published in 2019 and 2020 (4 and 8 articles, respectively). For the years 2015, 2017, and 2018, there was only one publication on this topic (Figure 2). Considering the quality criteria evaluated, the vast majority reached a low score in QC7 as presented in Figure 3.

Among these 15 articles in this group, we noticed that they focus on two of the three arboviral diseases transmitted by the *A. aegypti*. That is, 12 articles focused on dengue fever prediction models, whereas three of them focused on Zika virus disease. It is also important to highlight that, in all articles, the authors used machine learning algorithms in order to build their prediction model.

For Zika virus disease prediction, we noticed that the authors investigated different algorithms to predict positive cases of the disease. Jarrin et al. (73) evaluated support vector machines (SVM) and logistic regression to build their models, whereas Jarrin et al. (74) and Mahalakshmi and Suseendran (75) investigated Random Forest and Multilayer Percetron (MLP) algorithms, respectively.

Jarrin et al. (73) investigated SVM and RL algorithms—implemented in Python 3.7—to classify individual samples into “infected” or “uninfected” with the Zika virus. According to the author's results, the classifier showed a better accuracy for the “infected” class. The method presented by (73) can be used for the early diagnosis of ZIKV infection. Jarrin et al. (74), on the other hand, used mass spectrometry approaches to detect ZIKV by RT-PCR using RNA samples extracted from serum and urine to classify the diagnosis. The problem presented by (74) was modeled using Random Forest using MATLAB R2017a. The model presented by the authors is a robust platform that can be implemented in routine laboratories in order to help to support the diagnosis. Mahalakshmi and Suseendran (75) used the Multilayer Perceptron (MLP) artificial neural networks classifier. The data used is synthetic and was collected from the Internet. For prediction, the Weka software (version 3.8) was used. As the study was carried out with synthetic data, it is essential that tests be carried out with data from real databases. Having been trained only with artificial instances, when coming into contact with a real-world dataset, the accuracy of the generated model can drop significantly.

To predict dengue, the selected studies used different predictors. Mello-Román et al. (76) have developed a system in which data collection is based on the symptoms of the disease. The dataset is composed of cases registered by the Paraguayan health system, e.g., patients admitted due to fever and complete dengue diagnosis. Mello-Román et al. (76) carried out their tests using the IBM SPSS Modeler software in order to train their MLP and SVM algorithms. According to the author's results, the MLP showed better accuracy over the SVM classifiers.

(77) provide a prediction of the types of dengue cases. In order to investigate the best classifier, the authors evaluated the Decision Tree (DT) and Random Forest (RF) algorithms. Ho et al. (78) group explored a different method to speed up dengue diagnoses in the laboratory. The authors analyzed the decision tree (DT), deep neural network (DNN), and logistic algorithms. In this way, through the clinical parameters identified in the study, it is possible to help with the burden of laboratories for the diagnosis of dengue. Alam et al. (79) in their approach bring a prototype of a new framework for analyzing biomedical data called biocloud. The data gathered on this framework is modeled with a support vector machined to classify the disease's cases. This type of technology can provide services at a low cost and can be used in remote areas.

Ganthimathi et al. (80) developed an early dengue diagnosis system using Artificial Intelligence. In their research, Ganthimathi et al. (80) investigated two separate machine learning algorithms: support vector machine as well as k-nearest neighborhood. According to Ganthimathi et al. (80)'s findings, both algorithms presented good performances, however, the SVM showed superior performance compared to KNN's performance. Kapoor et al. (81) also treated the dengue prediction problem as a classification problem. Thus, in the study, Kapoor et al. (81) investigated four different classifiers, namely Random Tree, Random Forest, Support Vector Machine (SVM), and artificial neural networks. An interesting aspect of (81)'s model is that they used as their model's not only demographic information but also symptomatological data and clinical trial reports. Ariffin and Aris (82), in turn, created a system to help individuals in the self-diagnosis of dengue cases. As a classifier, the authors used artificial neural networks. As shown by the results obtained by (82), the model developed achieved high reliability for detecting the disease. Despite being a disease that helps in self-diagnosis, it is important to emphasize that medical guidelines are not dispensed with using the tool. Dharap and Raimbault (83) brought a different approach from the others studies commented so far. In their approach, Dharap and Raimbault (83) assessed the effectiveness of medical hematology analyzers that flags arboviruses' presence in blood samples. The machine learning algorithms used in their study were regression and Random Forest. With their results, they demonstrated that it is possible to screen arboviruses infection using a low cost, but also an effective predictor.

Srivastava et al. (84) bring a classification of dengue using online learning. Thus, learning takes place with just a few training examples. No retraining of the model or redeployment of the prediction engine is required. The following algorithms were used: Adaptive Regularization of Weights (AROW) and its Variants, Gradient Descent Online (OGD), Confidence Weighted Learning (CW) and Soft Variants (SCW 1 and Scw 2), Normalized HERD (NHERD), Passive Aggressive (PA) and its variants PA1 and PA2, Improved Ellipsoid Method (IELLIP), Approximate Large Margin Algorithm (ALMA), Second Order Perceptron and Perceptron (SOP), Relaxed Online Maximum Margin Algorithm (ROMMA), and Aggressive Romma (AROMMA). The evaluation of the classifiers was done offline in the Weka software with SVM and RF, and later, the classifiers were evaluated online. Additionally, this system is a health system helping to signal patients with a high probability of being diagnosed with dengue. Sasongko et al. (85) focused on finding the best backpropagation algorithms for early detection of dengue with the addition of multilayer perceptron (MLP) optimization through five algorithms. The backpropagation algorithms used were Gradient Descent (GD), BFGS Quasi-Newton (BQN), Conjugate Gradient Descent—Powel (CGD), Resilient Backpropagation (RB), and Levenberg Marquardt (LM). Additionally, the Levenberg Marquardt algorithm proved to be the best for detecting dengue. In other words, this algorithm solves the data outlier problem well.

Iqbal and Islam (86)'s group performed a performance evaluation of different dengue outbreak prediction classifiers. The methods were evaluated by eight different performance parameters. Iqbal and Islam (86) evaluated K-nearest neighbor (kNN), Support vector machine (SVM), Artificial neural network (ANN), Naive Bayes classifier, Decision tree, and Logistic regression classifier (LogitBoost) algorithms. The experiments were carried out with the Weka learning software. Among the trained algorithms, according to the authors, the one with the best performance was LogitBoost. This classifier had the best classification accuracy, sensitivity, and specificity metrics.

Balamurugan et al. (87) created a classifier for detecting dengue cases based on combinatorial characteristics based on weighted entropy scores based on ideal classification. The algorithms used to extract the most important attributes were Correlation based Feature Selection (CFS), Genetic Algorithms (AG) and Particle Swarm Optimization (PSO), in addition to the Optimized Classification Algorithm based on Weighted Entropy Score (EWSORA). Finally, the data were submitted to conventional classifiers such as Naïve Bayes, J48, Multilayer Perceptron (MLP), and Support Vector Machine (SVM). For evaluation, the Weka software was used. As metrics to evaluate the best models for predicting dengue, accuracy, true positive rate, precision, Recall, F Measure, and ROC were used. After applying the Genetic Algorithm (GA), Particle Swarm Algorithm (PSO), and Correlation-Based Resource Selector (CFS) algorithms for resource selection, the J48 and MLP classifiers proved to be better. EWSORA has greatly improved the accuracy performance for several classifiers, mainly for Genetic Algorithm (GA), Particle Swarm Algorithm (PSO), and Correlation Based Resource Selector (CFS).

### 3.3. Arboviruses Outbreaks and Risk Prediction

Among the studies evaluated in this systematic review, we observed that 18 articles were related to the prediction of the occurrence of arboviruses outbreaks, or the prediction of the risk of the occurrence of disease outbreaks (Table 2). Taking into account the years of publication of the articles, it is observed that most were published in 2016, as shown in Figure 2. Regarding the quality criteria, the studies achieved scores above 0.7, as shown in the graph in Figure 3. On the other hand, it is important to highlight that among the evaluated studies, the average QC7 score was quite low. In other words, most of the studies did not explicitly state the limitations of the investigated models.

Considering the types of arboviruses, we observe that the vast majority of the studies evaluated focused on the prediction of outbreaks or risk of dengue fever (18, 88–99). Two of the studies involving dengue risk prediction or dengue outbreak were focused on only one dengue serotype (dengue hemorrhagic fever) (89, 90). However, Brett and Rohani (95) show in their study an approach using each serotype for their prediction. In the study of (100) and (101), the authors investigated models for predicting the risk of Zika virus outbreaks, while (102) took into account all cases of arboviruses (dengue, Zika virus, and chikungunya) in their model.

As for the variables used to generate the prediction models, we observed that several studies included the climatic variables (90) where the most common are temperature (18, 91–94, 96, 98, 100, 102), rainfall (18, 91–94, 96–98, 100). Other climatic variables appear less frequently, such as wind speed (90, 91), vapor pressure (100), sunshine (91), atmospheric, and SST predictors (97). Regarding the predictors used for model generation, we observe a greater variety of predictors that are not associated with climate variables, when compared to the predictors used in Section 3.1. The population density, number of travelers, temperature, health expenditure per capita, gross domestic product per capita, water coverage, ZIKV transmission in nearby countries were examples of predictors used in the study of (100). In contrast, Akhtar et al. (101) used gross domestic product per capita, physicians per 1,000 people, and beds per 1,000 people, population densities, in addition to Zika cases. Predictors based on transmitter vector monitoring data have been extensively explored, such as mosquito occurrence (100), breteau index, and ovitrap index (98). It is important to highlight that two of the studies evaluated did not clarify which variables were used in the investigated prediction models.

Regarding the data sources, in a considerate amount of works, the authors usually obtained their databases through local government data sources (90–94, 96, 98, 99, 102). However, some works obtained their databases through other international sources, such as the US National Oceanic and Atmospheric Administration (95), Pan Amerian World Health Organization (PAHO), International Air Transport Associate, World Bank, US Bureau of Economic Analysis (101), and World Health Organization (WHO) (103). Two of the works included in this group did not present information related to the origins of the data sources obtained. In addition, no works were found that used alternative sources such as data originated by means of search engines, as well as data generated through social networks.

Finally, for risk predictions or prediction of arbovirus outbreaks, we found several approaches. Models were investigated using artificial neural networks (89, 101, 102, 104), decision trees (89, 99), gradient boosting regression tree (GBRT) (100), naïve Bayes (89), extreme learning machines (90), Least Absolute Shrinkage and Selection Operators (LASSO) and Ridge (92), support vector machines (SVM) (18, 91, 93). Moreover, early warning signals (EWs) derived from the theory of critical slowing down (95), the Shewhart model (98), population loss value at risk model (103) were also investigated.

In the articles evaluated for this group, we also observed that dengue was the arbovirus that received the most attention in terms of creating models for predicting outbreaks or disease risk. In the models of the studies, the climatic variables of temperature and precipitation are the predictors that appear most frequently in the prediction models. However, regarding predictors that are not related to climate variables, there is no assessment of which factors most impact the performance of the prediction model. That is, none of the studies presented the performance of models with different predictor configurations in a comparative way.

As for the types of models used, we observed that most studies used non-deep machine learning algorithms to generate the prediction models. Despite the promising results, it is difficult to indicate which algorithms had the best performances. The authors used different predictor configurations for the different models, which made it difficult to carry out a more in-depth analysis of the types of models used.

### 3.4. Models of Mosquitoes Dynamics, Breeding Sites Models

Of the 139 selected articles, 10 were predicted with vector control (Table 2). The years of publication range from 2015 to 2020. For the years 2015, 2016, 2017, 2018, 2019, and 2020, 1, 2, 1, 1, 4, and 1 were selected, in this order (Figure 2). Analyzing Figure 3, we found that the articles scored relatively low on quality criteria 5 and 7. Among these, 7 developed models based on machine learning and 5 based on statistical methods.

Haddawy et al. (105) featured a pipeline design to detect mosquitos' breeding sites using geotagged images with a machine learning approach. In Haddawy et al. (105)'s model, they use container count with resultant in order to create container density maps. The relationship between the densities of the eight types of recipients and the larval survey data was calculated using multivariate linear regression and obtained good precision. For object recognition, Haddawy et al. (105)'s group evaluated a convolutional neural network (R-CNN). Thus, creating geo-tagged container density maps is favorable for providing large-scale detailed hazard maps.

Raja et al. (106) developed early *Aedes* outbreaks prediction models using a machine learning approach. In order to build the prediction models, they used temperature, precipitation, start date notification, and notification date, as well as vector indices such as *Aedes albopictus, A. aegypti*, and larvae count.

Raja et al. (106) ran experiments using Bayesian Network Models method in order to create the prediction models. The system interface was implemented in C++ (backend) and the frontend implemented in JapaScript, CSS, and HTML5. The system is able to make predictions considering a 7 days horizon.

Asmai et al. (107)'s proposal was to create a Mobile Application for the Intelligent Detection of Mosquito Larvae (iMOLAP). The mobile app uses the convolutional neural networks (CNN) method, which is the Inception V3 model. The image that is captured is compared to a collection of predefined images to measure accuracy. Therefore, iMOLAP can classify Aedes and larvae species by imaging, and detecting the affected area of the site. This application can be a very important tool to assist in the surveillance and combat of mosquitoes. Lee et al. (108)'s, on the other hand, focused on developing a model to predict mosquito abundance. Thus, they considered climatic variables such as temperature, air humidity, wind speed, and precipitation as model predictors. The authors evaluated different approaches in order to build their model. They investigated using multiple linear regression (MLR), and artificial neural networks (ANN) algorithms. The correlation between climatic variables was assessed using the cross-correlation function. The metrics used were the correlation coefficients, the RMSE, and the agreement index. The results of models made with ANN were better than the MLR in all metrics. The approach brought by this study is interesting and can be very useful in mosquito monitoring. However, the authors did not describe well the apparatus necessary for collecting data on the number of mosquitoes. They did not describe the ANN configurations evaluated. In addition to not being described the number of tests to obtain a result with statistical significance.

In the study of (109), the authors developed a mosquito washing prediction system for *Aedes* in Recife, Brazil. The authors evaluated several types of regressors to build models to predict the number of properties with the presence of mosquitoes in Recife. Among the regressors, Extreme Learning Machines are Single Layer Feedforward Networks (SLFNs), Fuzzy Extreme Learning Machine, Bayesian Extreme Learning Machine, Interval Type-2 Radial Basis Function, Neural Network (IT2-RBFNN), and Online Extreme Learning Machine (OLEM). First, the spatial distribution of the number of properties that contained water containers contaminated with *Aedes* mosquito larvae was performed. Then, the spatial distribution of properties with mosquito larvae was performed and stratified by the type of water reservoir. Finally, the models are implemented on the real-time surveillance data. As metrics, percentage RMSE and training time were used. In this way, the prediction system shows the mosquito's hotspots. This study takes a spatiotemporal approach, so research can help managers by giving direction to location-based mosquito population control policies, helping to limit transmission to humans.

Bennett et al. (110) brought a mosquito classification to detect *A. aegypti*. The database was created by the authors themselves. They collected samples of larvae present in garages that traded used tires in Panama. Additionally, with mass spectrometry, the types of larvae are identified. Finally, using the Supervised Neural Network (SNN) a classifier is built to identify the type of mosquito present. The model created had a very high capacity for recognizing and classifying training data. This study brings a look at the garages, which can be a strategic point for epidemiological surveillance policies.

Considering the statistical models, we highlight the study of (111). Their group has developed time prediction models for *A. aegypti* oviposition. Both model validation and application were applied in the dengue outbreak in 2016. For this purpose, time series of MODIS (moderate resolution image spectroradiometer sensor) products of normalized difference vegetation index and daytime surface temperature were created. The MODIS model consists of: (1) linear regression modeling and (2) the creation of two models, one with and one without lag times on the independent environmental variables. The environmental variables were standardized and the developed models were compared using the Akaike Information Criteria (AIC) to determine the ideal model in terms of goodness of fit and number of parameters. The model without latency was the best. Both models developed in this article showed that MODIS environmental variables (NDVI and LST) are good predictors because both environmental variables are present in both models, providing acceptable fit and validation results. We can understand that the NDVI increment may be due to precipitation in the near past followed by an increment in the vector activity which is verified by the increments of the oviposition activity. Furthermore, a model based on MODIS has the possibility to envision an operational forecast program at national level.

Estallo et al. (112) created a prediction model evaluating the weather variability associated with the seasonal fluctuation of the oviposition dynamics of *A. aegypti* in a City of Orán, Argentina. To create the model, precipitation data, photoperiod, water vapor pressure, temperature and relative humidity (maximum and minimum) and ovitrap sampling were used. A multiple linear regression analysis was performed with the set of meteorological variables considering the time lag that correlates with oviposition. And the model is validated. The prediction model created allows the prediction of the growth or decrease of the ovitraps activities of *A. aegypti* based on meteorological data. The prediction of these activities can be predicted three or 4 weeks in advance. Because this model brings a more localized and comprehensive assessment with site-specific data that can be used in disease prevention policies.

Hettiarachchige et al. (113) built a data transmission risk prediction model based on high resolution meteorological data. Additionally, this risk is predicted through vector prediction. Routine entomological surveillance data for dengue and meteorological data from a prediction system with high spatial and temporal resolution were used. The risk prediction system was divided into two stages to assess dengue transmission *via A. aegypti*. In the first, logistic regression was used to determine the presence or absence of larvae in the sites of interest using climatic attributes as explanatory variables, and then used a bootstrap approach in an administrative division. In the second, with the negative binomial model inflated to zero, an estimate of the larvae count of the positive division predicted in the first stage is made, and then positive larvae sites are identified and the number of larvae is predicted. Splitting the model into two stages increases the accuracy of identifying positive larval locations. A benefit for risk prediction in non-homogeneous regions.

da Cruz Ferreira et al. (19) developed a temporal prediction of mosquito infestation based on climatic data and monitoring data from *Aedes*. The climatic variables used were daily rain, temperature (minimum, average, and maximum), and relative humidity, and dengue data were obtained from the Health Department of Porto Alegre. The Generalized Additive Model (GAM) and Logistic Regression methods were used. The first method was used for two models, one was fitted with climatic variables, and the other with climatic variables and mosquito abundance as an explanatory variable. Additionally, the second method was used to assess the effect of adult mosquito infestation on the probability of dengue incidence. The second GAM model predicted the data better than the first. The researchers stated that if the population of *Aedes* is continuously monitored the predictions of the infestation rate will be more reliable. And monitoring this population is important for dengue control in Brazilian cities.

The studies presented here brought several different perspectives to control the *A. albopictus* and *A. aegypti* mosquitoes. Some of the variables considered in these studies were: stratification by type of water reservoir; neglected environments, such as garages that contain tires and other potential breeding sites; local and comprehensive assessment of breeding sites; evaluation of mosquito larvae stages; and the seasonality of the mosquito cycle dynamics. Furthermore, in the construction of the prediction models, different machine learning techniques and statistical methods were used. The models with a broad and more restricted evaluation of the study regions proved to be good and robust in terms of evaluation metrics. Many of these works present scalability and reproducibility for prediction at the national level, relating the magnitude of the population of Aedes mosquitoes, the incidence of arboviruses, and the monitoring of this vector. Thus, these approaches can be used to support the implementation of epidemiological surveillance policies. However, some of these studies had limitations, the lack of clarity and uniformity regarding the evaluation metrics and the number of tests, in order to obtain results with statistical significance. Some of these studies also omitted the complete description of the configurations of the adopted classifiers.

### 3.5. Clustering, Spatiotemporal Modeling

Prediction models involving clustering and spatiotemporal prediction presented relatively few studies when compared to the other approaches presented in this systematic review (Table 2). For studies with only these types of approaches, the year with the highest production was 2015, when 5 articles were published on the theme (Figure 2). An important point to highlight is the fact that, in the studies included in this group, the authors achieved the highest scores regarding the quality criterion involving the discussion about the limitations presented by the models (Figure 3).

Mathur et al. (114) brought a spatiotemporal prediction of dengue. This study also discussed and implemented dengue modeling with clustered incidence map visualization in Selangor, Malaysia. The spatiotemporal mapping was performed using the clustering technique with the k-mean algorithm. Thus were generated the incidence clusters. Then, the Gaussian mixture model was applied, finding the incidence density of dengue. Next, the K-means (K-NN algorithm) was used to find the centroid of the incidence. The Expectation-Maximization (EM) Algorithm was used to relate the clusters. The Bayesian Information Criteria (BIC) is then used to optimize the EM. Finally, with the Geographic Information System (GIS) technique, it is possible to accurately visualize the mapping of dengue incidence vulnerability in Selangor. The latter was used in the prediction. To create the proposed model, the R studio software was used, and to measure the vulnerability index, the K-means grouping was used. This study brings a spatiotemporal approach that can be used to implement health promotion policies. Another study that brought the spatiotemporal approach was the work of (115). In his study, Andersson et al. (115) made use of street images (Google Street) to implement a model to predict dengue hemorrhagic rates in the city of Rio de Janeiro, Brazil. In order to create this model, a siamese convolutional neural network technique was used. First, to create the models, dengue data in Rio de Janeiro were obtained and normalized. Next, street images were labeled according to latitude and longitude. The capacity of convolutional neural networks was analyzed with two approaches Simple-4CSCNN and ResNet-4CSCNN. The proposed models were implemented in the PyTorch framework. Simple-4CSCNN proved faster, with better loss of rating, but exhibited worse results in the validation test set. ResNet-4CSCNN generalized the training data well and reasonable results in the test set. The advantage of this approach is the use of street images to predict dengue cases, and the lack of work on the same line makes comparisons difficult.

The study of (116) was aimed at mapping the probability of an epidemic outbreak of Zika in the world. For this, three models were implemented, reverse propagation neural network (BPNN) (with sigmoid activation function), gradient increase machine (GBM), and random forest (RF). High-dimensional multidisciplinary covariate layers were combined with comprehensive localization data on Zika virus infection in humans. In addition to the demographic distribution data of the *Aedes* mosquitoes, global climate data, socioeconomic data, night light data, and human movement data were used. To create the models, the R language (version 3.3.3) was used. Models were trained with cross-validation 10 times. To assess the performance of the prediction models, the ROC curve was used. The models created were robust and capable of simulating the global probability of transmission risk of ZIKV and also quantified the uncertainty of the accuracy of the prediction models. The models created provided reference information for model selection in the area of epidemiological cartography. However, the study only uses the AUC as a metric for evaluating the models.

In the study of (117), the authors developed a model for clustering and mapping dengue risk susceptibility. In his model, Ghosh et al. (117) used as variables epidemiological data, temperature (maximum and minimum), precipitation, relative humidity and Earth Surface Temperature (LST) images, demographic, socioeconomic, vegetation, and water index data. Two statistical methods were used to create the models: Poisson Models (to form the clusters) and Multiple Logistic Regression. This first was used to estimate the incidence of dengue. Moran location and weighting function I based on the specific spatial distance of the outlier were also used. This second function was used to estimate the probability of dengue occurrence using climatic variables as attributes. The researchers observed a strong association between monthly dengue cases and monthly mean rainfall and an association between monthly mean air humidity and disease cases. The model takes into account a spatiotemporal approach for predicting dengue risk. In addition, it considers the social and demographic aspects of predicting dengue.

In (118)'s approach, the authors created spatiotemporal prediction models for dengue cases taking into account population density. As variables, dengue cases in the city of Khyber Pakhtunkhwa, transmission vector records (*A. aegypti* and *A. albopictus)*, population density and distance to roads and rivers were considered. As methods, logistic regression, variogram function, and binomial kriging with a binary logistic drift were used. Logistic regression was used to assess the correlation between dengue cases and other variables (covariants). Then the variogram function (spherical, Gaussian, circular, and Matém) is calculated for the city under study and its subregions. Additionally, at the end, the estimation of the weights of the kriging equations is done using the weights of the variogram model. The researchers claim that the “presence” of the mosquito and population density affect the dynamics of the disease. And the models performed well in cities with high population density. However, the study did not make clear the databases used as well as the periods chosen for modeling, testing, and validation.

Phanitchat et al. (119) developed an identification of sub-district level dengue clusters in Thailand. For this purpose, data on weekly dengue cases (by gender), population density per *Km*^2^ temperature, and rain in the same period for Khon Kaem province were used. The models used were Bayesian Poisson Regression and Local Indicators of Spatial Association (LISA). The first was to assess the relationship between the number of monthly dengue cases in the 199 sub-districts. The metric for evaluating the fit of the model was the Wantabe-Akaike Information Criterion (WAIC). Finally, LISA was used to identify hot and cold spots and outliers in the incidence of dengue. The article concludes that dengue outbreaks are more frequent in the rainy season. With the analysis by hotposts, it is observed that there is a cluster of cases around the urban areas of Khon Kaem and in rural areas in the southwest of the region. The spatiotemporal approach is useful for application in health promotion strategies. However, there is an inherent limitation regarding the collection of public data, such as underreporting of cases, errors in reporting symptomatic cases, and absence of asymptomatic cases. In addition, the use of data is a little out of date.

Chen et al. (120) developed a new framework for producing spatiotemporal prediction at the neighborhood level. Various data were used, such as dengue incidence data (with home address data and start date), movement patterns, construction age of buildings in a neighborhood, meteorological data (maximum and minimum temperature and average relative humidity), number of national weekly cases, index by Normalized Difference (NDVI) among others. The separate prediction models and submodels created were based on LASSO for each prediction window. Climatic variables and their effects have a greater effect when analyzing longer time intervals. The fact of having less vegetation, older buildings, greater connectivity to other areas, and more travelers arriving in the area causes the number of cases to increase. The proposed model brings a spatiotemporal approach at the neighborhood level up to 3 months in advance. The system proved to be robust to changes in baseline incidence over time.

Jat and Mala (121), in turn, brought an approach to the use of digital geospatial technologies to identify potential sources of dengue incidence. For this purpose, the spatiotemporal grouping of dengue incidences was performed using the Kulldorff scanning method. With the help of Getis-OrdGi statistics, high-risk areas were identified and then implemented in the GIS. And the data obtained was correlated with meteorological parameters, such as wind speed, humidity and demographic factors, such as age and gender. This work shows that the occurrence of dengue is not random, it is directly linked to meteorological phenomena. Thus, this study serves as a warning and to use actions to group regions that may be focuses on dengue spread.

The studies cited here brought interesting approaches to dengue and Zika, considering both epidemiological and climatic data (such as precipitation and temperature), as well as population density, age of construction of buildings, socioeconomic and demographic data, and cases of the disease by patient gender. One of the works is the first, as far as is known, to use street view images to predict dengue cases, something quite innovative. The models created had good results regarding their evaluation metrics. Both machine learning models and statistical models were used. These surveys also rely on algorithms that do not have a great computational weight, which makes their use by the public service viable. The models made were both at the sub-district level and the global level. The spatio-temporal approach brought by the studies in this section helps health managers in directing public resources to areas that need more attention.

### 3.6. Other Approaches

The articles included in group 6 are articles that combined more than one approach in their predictions, or that had a very different approach from the rest of the articles evaluated in this review. According to Figure 2, three of the studies were published in the year 2019. In the years 2016, 2017, and 2020, 1, 1, and 2 studies were published, respectively (Figure 2). Analyzing the scores referring to the quality criteria, the average scores in most QC were above 0.7, except QC5 and QC6 (Figure 3).

Among the seven articles, three of them simultaneously addressed numerical prediction models of arboviruses cases and also prediction of the risk of epidemiological outbreaks (122–124). In the study of (125), the authors addressed risk production models as well as clustering models to identify regions with similar patterns of disease transmission. Harumy et al. (126), on the other hand, the authors investigated prediction models of the area as the greatest potential to suspend arboviruses and case prediction. Yamamoto et al. (127), in turn, brought an approach to detecting the importation of arboviruses into a country. As for arboviruses groups, the publications were mostly concentrated on dengue (122, 124, 125). Only Yamamoto et al. (127) brought a study considering Zika virus disease cases.

It is important to highlight the variety of models that were covered. Among them, we can mention Random Forest, RF-USA, Logistic Regression (124), and Naïve Bayes (125) for the classification steps. Both (123) and (122) considered a threshold value for identifying an epidemic or outbreak. In the steps involving regression, probabilistic models (127), LASSO, ARIMA, SARIMA (124), Generalized Linear Regression (123), Artificial Neural Network (122, 126), SEIR model (122), and multiple variate regression (125) models were used.

For studies that presented a mixed approach, models for the numerical prediction of cases are essential for the analysis of the epidemiological curve of the disease. In this way, health authorities may have indications that combat policies are or are not effective in combating arboviruses. On the other hand, predictions with spatial approaches can indicate regions with more or less intensity of cases, which can help the distribution of financial and human resources to the most critical regions. Therefore, a mixed approach to the prediction of arboviruses is shown to be robust to assist in decision-making on arbovirus prevention policies.

## 4. Conclusion

Arboviruses have a major impact on populations affected by seasonal outbreaks of these diseases. In addition to the impact caused by the number of deaths and infections, the socioeconomic impacts tend to remain until the next outbreak. The prevention and control of the occurrence of these diseases are directly associated with the monitoring/control of their transmitting vector.

In this sense, this systematic review aimed to identify predictive models of diseases transmitted by *A. aegypti*, as well as identify existing models for modeling vector dynamics. For this, we defined a review protocol that was followed throughout the process. We obtained 429 publications retrieved from scientific databases using a predefined search string. After filtering through the exclusion and inclusion criteria, 139 studies remained in the review for analysis and evaluation of quality criteria.

The remaining studies after the entire analysis process were grouped according to their similar characteristics. Arboviruses' prediction studies are mostly linked to the numerical prediction of cases. According to the results obtained, we observed that among the arboviruses transmitted by *A. aegypti*, most of the studies are aimed at predicting dengue. Both in numerical prediction models, as a prediction of outbreaks, epidemics, and disease diagnosis. Studies regarding predictions with a spatiotemporal approach are also more focused on dengue rather than on Zika and chikungunya. An important point to highlight is the fact that few studies were focused on the spatiotemporal prediction of diseases, as well as the prediction of models related to mosquito dynamics.

Another point that can be highlighted in the studies in this review is in relation to the variables selected for the generation of arbovirus models. In the case of modeling taking into account numerical prediction, prediction of outbreaks and epidemics, and spatiotemporal prediction, we observed that most studies consider climatic variables as model parameters. Among them, the most common are the historical series temperature, rain and relative humidity. However, parameters related to natural phenomena and also variables obtained by remote sensing also gained prominence, as well as data from social networks and search queries. Furthermore, data related to vector monitoring have also been included both in numerical prediction models of arboviruses and in models related to the dynamics of the *A. aegypti* itself. On the other hand, in arbovirus models that prioritize the detection of infection in the individual, we note that the most used parameters are symptomatological parameters. The use of models based only on symptomatological parameters can cause fever-like diseases to be confused with dengue, Zika, and chikungunya. However, we also found studies that use hematological parameters to detect infection.

In this study, we analyzed that, for prediction problems involving arboviruses and also involving mosquito dynamics, a large part of the data is obtained through local health and climatology agencies. Missing data and cases of underreporting by health agencies are one of the most reported problems in the studies evaluated.

Furthermore, this systematic review also demonstrated that there is a range of models that are widely used in prediction problems. Poisson models and moving average models (ARIMA, SARIMA) are widely used to predict historical series. However, we observe that artificial neural networks, support vector machines, and decision tree-based models are widely explored by the studies in this review. It is important to highlight that in many of the works that use Artificial Intelligence models, the authors often do not describe the configurations of the evaluated models and how the models were validated. In other words, although the models have good evaluation metrics, there is no way to guarantee their statistical relevance.

Finally, the arboviruses dynamics is a very heterogeneous problem that involves the interaction of various factors such as climatic and environmental factors, mosquitoes, and human beings. The heterogeneity of arbovirus dynamics is precisely what makes the prediction problem a very complex problem. Therefore, from this systematic review, we hope to provide a theoretical foundation regarding the state-of-the-art of dengue, Zika, and chikungunya prediction models, as well as the breeding sites of its main urban transmitter vector. Hence, we believe that there is great potential for exploring models with a spatiotemporal approach. These models can be an important tool in the fight against arbovirus-borne diseases, as they contain spatial information of epidemiological interest that will be able to more effectively direct human and financial resources, especially in more vulnerable countries.

## Data Availability Statement

The data and materials for all experiments reviewed in this study are publicly available as open datasets and are cited in the body of the text.

## Author Contributions

CL, AnS, GM, CC, AbS, and WS designed the research protocol. CL, AnS, GM, and CC wrote the document review. AM, AA, LD, IB, MT, EB, and SB supported the research. AbS, TM, TA, LC, OY, PK, KJ, and WS supervised and supported all the work. All authors contributed to the article and approved the submitted version.

## Funding

This study was funded by the Brazilian research agencies FACEPE, CAPES, CNPq, and the University College London held UKRI research grant number NE/T013664/1.

## Conflict of Interest

The authors declare that the research was conducted in the absence of any commercial or financial relationships that could be construed as a potential conflict of interest.

## Publisher's Note

All claims expressed in this article are solely those of the authors and do not necessarily represent those of their affiliated organizations, or those of the publisher, the editors and the reviewers. Any product that may be evaluated in this article, or claim that may be made by its manufacturer, is not guaranteed or endorsed by the publisher.
